# YouTube-videos for patient education in lymphangioleiomyomatosis?

**DOI:** 10.1186/s12931-022-02022-9

**Published:** 2022-04-27

**Authors:** Finn M. Wilkens, Claudia Ganter, Katharina Kriegsmann, Heinrike Wilkens, Nicolas Kahn, Gillian C. Goobie, Christopher J. Ryerson, Michael Kreuter

**Affiliations:** 1 Center for Interstitial and Rare Lung Diseases, Dep. Pneumology, Thoraxklinik, University of Heidelberg, Heidelberg, Germany; 2 German Center for Lung Research, Heidelberg, Germany; 3 Department for Hematology, Oncology and Rheumatology, University of Heidelberg, Heidelberg, Germany; 4 Department of Pneumology, Allergology and Critical Care Medicine, University Medical Centre, Saarland University, Homburg/Saar, Germany; 5 Clinician Investigator Program, Department of Medicine, University of British Columbia, Vancouver, BC Canada; 6 Department of Human Genetics, Graduate School of Public Health, University of Pittsburgh, Pittsburgh, PA USA; 7 Simmons Center for Interstitial Lung Disease, Division of Pulmonary, Allergy and Critical Care Medicine, University of Pittsburgh, Pittsburgh, PA USA; 8 Department of Medicine, University of British Columbia and St. Paul’s Hospital, Vancouver, BC Canada; 9 Centre for Heart Lung Innovation, University of British Columbia and St. Paul’s Hospital, Vancouver, BC Canada

**Keywords:** Lymphangioleiomyomatosis, Patient education, Video, DISCERN, HONcode

## Abstract

**Background:**

The Internet is commonly used by patients to acquire health information. To date, no studies have evaluated the quality of information available on YouTube regarding lymphangioleiomyomatosis (LAM). Our aim was to determine the quality and content of YouTube videos regarding LAM and to compare the information provided with current knowledge and guidelines about the disease.

**Methods:**

The first 200 video hits on YouTube in English for the search term “lymphangioleiomyomatosis” were recorded. All videos suitable for patient education on LAM were included. Video quality was analyzed independently by two investigators utilizing the Health on the Net (HONcode) score, which assesses whether websites provide understandable, accessible, and trustworthy health information; the DISCERN score, which evaluates the quality of information about treatment decisions; and a newly developed LAM-related content score (LRCS) with 31 guideline elements.

**Results:**

The search identified 64 eligible videos. The “engagement rate” of 0.3 was low, with a median number of views of 408 (range 42–73,943), a median of 4 likes (range 0–2082), and the majority (53%) receiving a low HONcode score (≤ 2) and only 10% of videos achieving a high score (> 5). The median DISCERN score was 28 (range 15–61, maximum possible score 80), indicating poor video quality and reliability. The median LRCS was 8 (range 0–29, maximum possible score 31) and videos frequently failed to provide sources of information.

**Conclusions:**

Online resources could contribute to the limited and often inaccurate information available to patients with LAM, with only a few YouTube videos providing high-quality patient-relevant information.

**Supplementary Information:**

The online version contains supplementary material available at 10.1186/s12931-022-02022-9.

## Background

Lymphangioleiomyomatosis (LAM) is a rare and incurable lung disease that occurs mainly in women as either sporadic (S-LAM) or tuberous sclerosis complex-associated LAM (TSC-LAM) [1, 2]. LAM results from a mutation in the tuberous sclerosis complex 1 or 2 genes (TSC1, TSC2) that control cell growth, resulting in pulmonary cyst development caused by proliferation of abnormal smooth muscle cells that migrate into the lung and displace normal lung tissue [3]. Other organs such as the kidneys may also be affected. In LAM, data from the NHLBI LAM Registry estimate a median transplant-free survival of over 20 years after diagnosis [4]; however, almost all patients suffer from slowly worsening shortness of breath with progressive loss of lung function and limitation of gas exchange [5–7]. The prevalence of sporadic LAM is unknown, with estimates ranging from 3 to 8 per million women [3].

Despite existing guidelines [8], physicians are often poorly informed about pathophysiology and treatment options for LAM due to the rarity of the disease, and affected patients often rely on actively seeking additional information on their own [9]. Over the past 10 years, social media has become an important resource for patients seeking health-related information [10, 11]. YouTube is the most widely used video platform and the website domain with the second most views in the world after Google [12]. YouTube is increasingly consulted for health-related questions [13]. However, a previous study on idiopathic pulmonary fibrosis (IPF), another rare lung disease, revealed that the information regarding IPF on YouTube was of low quality [14], with another study by the same group indicating that patients with IPF were unable to obtain quality information regarding their disease on the Internet [15].

The objectives of this study were to analyze the content and quality of the most frequently accessed YouTube videos on the topic of LAM, and to compare the content of these videos to clinical practice guidelines [3, 8]. We hypothesized that the information contained in YouTube videos regarding LAM would not provide accurate and reliable information to affected patients and that videos would have important information deficits.

## Methods

### Study overview

This was a retrospective systematic assessment of YouTube videos about LAM that were targeted to patients.

This work was part of an investigation in LAM with patient involvement. An ethics approval was obtained accordingly (EK Number S-758/2020).

### Search strategy

On the cut-off date of April 20, 2020, the first 200 video hits on YouTube were recorded for the search term “lymphangioleiomyomatosis”. The search was performed on a Windows system (Microsoft Windows 10, Lenovo PC) with a newly installed browser (Mozilla Firefox) after deleting the browser history and cookies to avoid influencing the results by previous searches. USA was chosen as the location of the English-language search. Videos were downloaded and saved. Any video containing information for laypersons suitable for patient education about LAM was included, which required that a video includes at least one of the following: definition, symptoms, risk factors, diagnostic tests, management, and/or long-term sequelae. In the case of a video playlist with multiple videos, only the first video in each case was included, unless it was a playlist with consecutive chapters on LAM education. Videos were excluded if they were presented in a language other than English, were duplicates or videos without audio. If inclusion or exclusion criteria were not clearly met, the video was re-evaluated by the second reviewer (HW) and a consensus decision was made.

### Video evaluation

The metadata of each video (URL, title, upload date, country of origin, duration of the video, likes, dislikes, number of views, and comments) were recorded for each video when available. The “viewing rate” (number of views/number of days since upload × 100) and “engagement rate” ([number of likes + dislikes + comments]/number of views × 100) were calculated for each video [16]. Videos were categorized into four main groups: (1) academic institutions or government organization, (2) news media organizations or journalistic outlets, (3) independent medical professionals or users, or (4) independent non-medical users. All videos could be categorized into one of the four groups. Two investigators (FW and HW) independently evaluated each video for content and quality using the DISCERN, HONcode, and content scores, based on both audio and visual information. Disagreements among investigators regarding the classification or rating of a particular video were discussed until consensus was reached.

The DISCERN instrument is a validated set of 16 questions used to assess the quality of written consumer information about treatment decisions, with each answer ranging from 1 (severe deficits in fulfilling quality criteria) to 5 (quality criteria of completely fulfilled) [17] (Additional file 1: Table S1). In contrast to an expert focused tool, it is a user focused tool for quality of consumer health information to measure acceptable levels of reliability and validity [17]. This instrument has been previously adapted to evaluate video information [14]. HONcode (Health on Net Foundation Code of Conduct) provides an ethical standard for objective and transparent assessment of medical information available on the internet [18, 19]. It consists of eight questions on authority, complementarity, privacy, attribution, justification, contact details, financial disclosure, and advertising policy (Additional file 1: Table S2). For each domain, the videos were rated as either 0 if criteria were not met or 1 if criteria were met. HONcode scores between 0 and 2 were ranked as low, 3–5 as medium and 6–8 as high. The LAM-related content score (LRCS) was developed using 31 previously defined guideline elements related to the diagnosis, treatment, and prognosis of LAM [8, 20]. The score was grouped into definition, complications, symptoms, diagnostics, risk factors, treatment, and long-term sequelae. An additional question was asked regarding information about COVID-19.

### Statistical analysis

Statistical analysis was performed using R (R version 4.1.1).

Data are presented as absolute numbers and percentages, and medians and ranges (minimum–maximum) as appropriate.

Chi-squared tests were used to analyze contingency table distributions. Between-group comparisons were made using t-tests, Wilcoxon rank sum tests, ANOVA, or Kruskal–Wallis H tests, as appropriate. Multiple linear regression was performed to evaluate the association of video characteristics (search rank, viewing rate, engagement rate) with both quality (HONcode score and total DISCERN score) and content (sum of the LRCS). Unsupervised hierarchical clustering (R package ‘ComplexHeatmap’) by video and HON code score, DISCERN, and LRCS items was performed to identify associations between website category and video quality or content. A p ≤ 0.05 was considered statistically significant for all analyses.

## Results

### Video characteristics

**Fig. 1:**
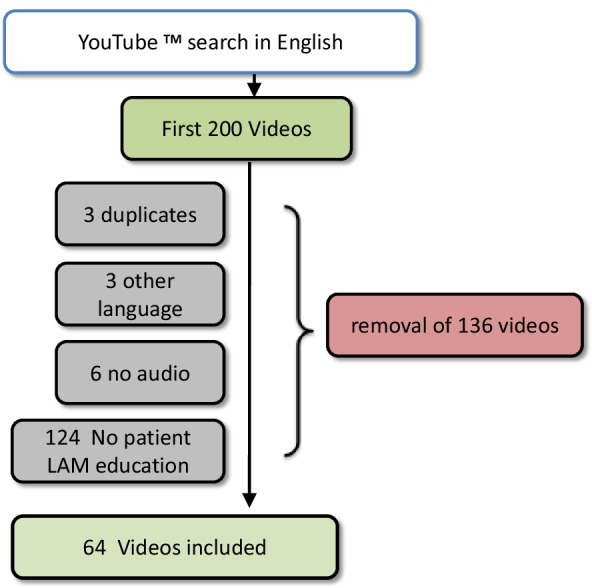
Flow diagram of YouTube Video selection. Reasons for exclusion of videos are listed on the left side of the arrow

The video selection process is shown in Fig. 1. In the English-language search, 64 of 200 videos met eligibility criteria. The median time between video upload and analysis was 49 months (range 0 to 148). Videos had a median of 408 views (range 42 to 73,943), with all analyzed videos together being viewed 129,923 times (Table 1). Two thirds (67%) of videos were from North America, 20% from Europe, and 13% from Australia.

**Table 1:** Characterization of unique videos

Overall unique videos, n (%)	64 (100)
Video category, n (%)	
Academic Institution	14 (22)
Governmental organization	1 (2)
News/media	6 (9)
Industry/for-profit organization	0 (0)
Independent medical professional	5 (8)
Independent non-medical user	38 (59)
Host continent, n (%)	
Europe	13 (20)
North America	43 (67)
Australia	8 (13)
Video duration, minutes	
Median (range)	5 min 7 s (20 s–372 min 28 s)
Time in months since upload, median (range)	49 (0–148)
Views, median (range)	408 (42–73,943)
Likes, median (range)	4 (0–2082)
Dislikes, median (range)	0 (0–36)
Comments, median (range)	0 (0–1151)
Viewing rate, median (range)	0.7 (0.02–532)
Engagement rate, median (range)	0.01 (0.00–0.79)

The number of views were higher for videos from academic institutions and governmental organizations (median 1629; range 150 to 4420) and news or media (mean 938; range 73 to 73,943) than from independent medical professional (mean 364; range 302 to 966) or independent nonmedical users (mean 306; range 42 to 6479) (p = 0.02) (Table 2). Videos had a median number of likes of 4 (range 0 to 2082) and dislikes of 0 (range 0–36). Across all videos, the viewing rate averaged 9.14 ± 66.40 with a median engagement rate of 0.01 (range 0.00 to 0.79), without significant differences between the four groups.

**Table 2:** Characterization of unique English videos by category

Video category	Academic institution/Governmental organization	News/media	Independent medical professional	Independent non-medical user	P value
Videos, n	15	6	5	38	/
Host continent, n (%)					
Europe	1 (7)	1 (17)	1 (20)	10 (26)	0.008^a^
North America	13 (87)	5 (83)	3 (60)	22 (58)	
Australia	1 (7)	0 (0)	1 (20)	6 (16)	
Video duration, Median (range)	4 min 25 s (35 s–37 min 29 s)	2 min 50 s (58 s–59 min 59 s)	9 min 4 s (5 min 8 s–55 min 07 s)	6 min 42 s (20 s–6 h 12 min 28 s)	0.199
Time in months since upload, median (range)	53 (3–109)	64 (4–108)	46 (12–66)	49 (0–148)	
Views, median (range)	1629 (150–4420)	938 (73–73,943)	364 (302–966)	306 (42–6479)	*0.023*
Likes, median (range)	11 (1–25)	7 (0–2082)	5 (1–48)	3 (0–40)	0.294
Dislikes, median (range)	0 (0–1)	0 (0–36)	0 (0–0)	0 (0–5)	0.883
Comments, median (range)	1 (0–20)	1 (0–1151)	0 (0–10)	0 (0–21)	0.123
Viewing rate, median (range)	1.30 (0.13–7.46)	0.30 (0.02–532)	0.21 (0.18–2.63)	0.23 (0.05–2.69)	0.487
Engagement rate, median (range)	0.01 (0.00–0.06)	0.01 (0.00–0.07)	0.02 (0.00–0.06)	0.01 (0.00–0.79)	0.984

### Video quality evaluation with DISCERN scores and HONcode

**Fig. 2:**
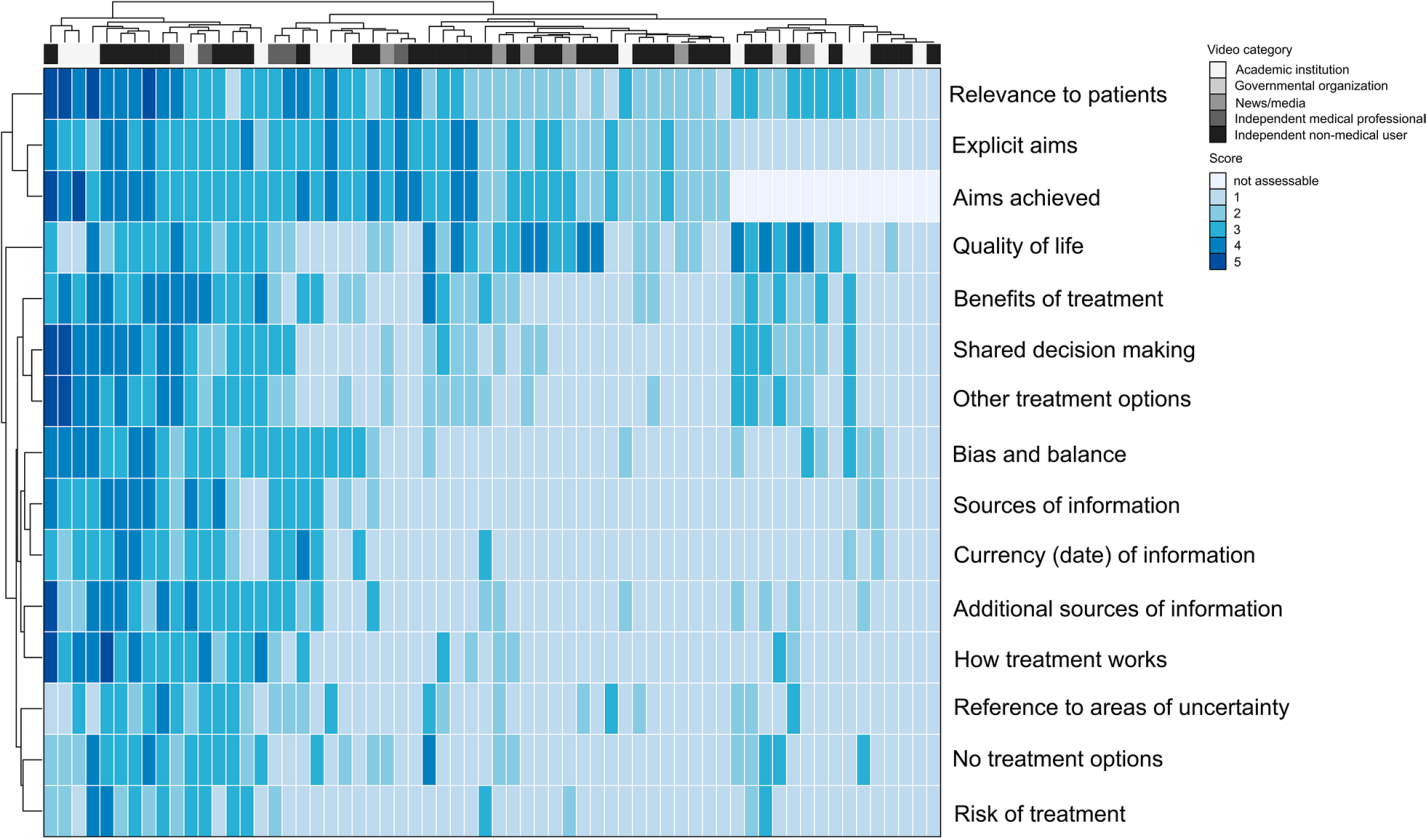
DISCERN score for English videos. Unsupervised hierarchical clustering was performed by DISCERN score items (rows) and single videos (columns, n = 64). The categorial item scoring ranges between 1 (not addressed/fulfilled) and 5 (fully addressed/fulfilled). The item 2. “aims achieved” was not assessable (NA) scores, when item 1. “Explicit aims” was scored with 1, i.e., criterion not met. The video category is shown in the top row of the heatmap

Videos were assessed for user-focused video quality using the DISCERN instrument, where the average total score was 2 (range 1 to 5). Unsupervised hierarchical clustering demonstrated that few videos achieved high scores, especially for questions related to treatment and risks of therapy, further resources for information, and publication date (Fig. 2). Additional file 1: Fig. S2 shows items for single videos. Videos produced by academic institutions or governmental organizations and independent medical professionals generally had higher median DISCERN scores (median 30; range 15 to 57; median 40; range 26 to 48, respectively), compared to videos provided by news or media (median 26; range 20 to 28) or by independent non-medical users (median 28; range 15 to 61); however, this was not statistically significant (p = 0.17) (Table 3).

**Table 3:** Characterization of unique English videos by category for HON score, DISCERN score and LAM-related content score

Video category	Academic institution/Governmental organization	News/media	Independent medical professional	Independent non-medical user	P-value
	N = 15	N = 6	N = 5	N = 38	
HON score					
Median (range)	3 (2–6)	2 (0–2)	4 (2–6)	2 (0–7)	0.058
Rating according to HON foundation score, n (%)					
Low	5 (33)	6 (100)	1 (20)	22 (58)	0.063^b^
Medium	9 (60)	0 (0)	2 (40)	9 (24)	
High	1 (7)	0 (0)	2 (40)	7 (18)	
Sum DISCERN score					
Median (range)	30 (15–57)	26 (20–28)	40 (26–48)	28 (15–61)	0.173
Sum LAM-related content score					
Median (range)	9 (0–25)	7 (4–15)	10 (8–13)	6 (0–29)	0.185

*HON* Health on the Net, *SD* standard deviation

^a^North America and Australia were grouped and compared to Europe as host continent

^b^Category “news/media” was excluded from statistical analysis, as none of the videos were ranked as medium or high

**Fig. 3:**
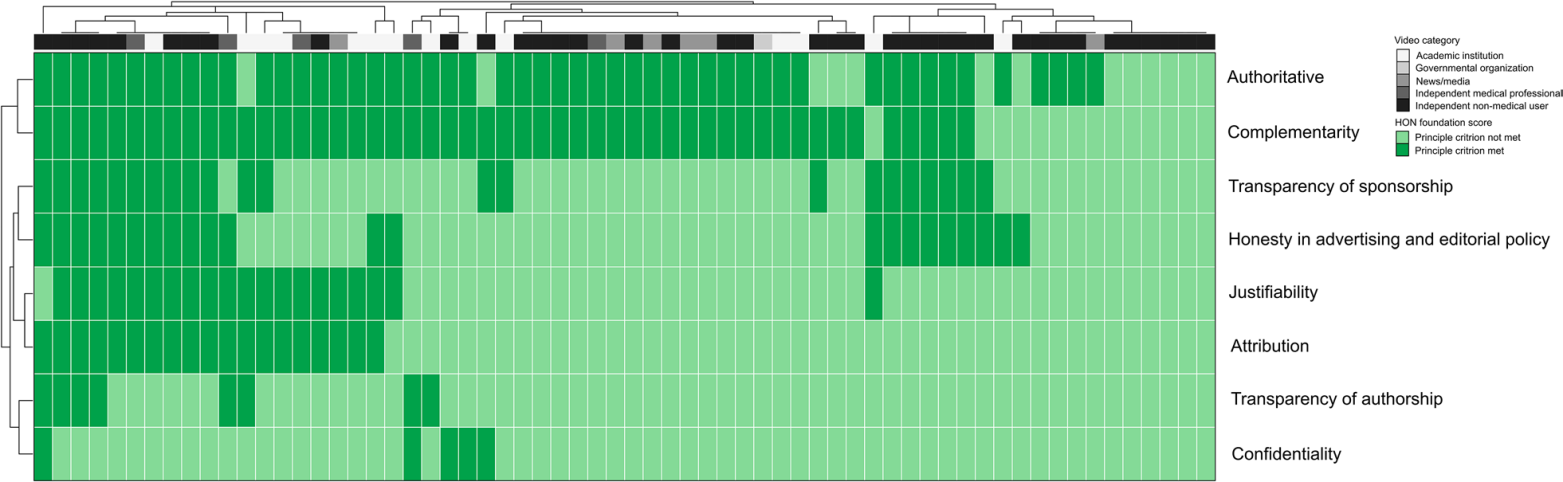
HON foundation score for English videos. Unsupervised hierarchical clustering was performed by HON foundation score items (rows) and single videos (columns, n = 64). The video category is shown in the top row of the heatmap

Using the HONcode score to assess general video quality, the criteria authoritativeness and complementarity were met in 80% and 78% of the videos. Each of the other six HONcode principles were fulfilled in less than 35% of videos. Source data was not referenced in 70% of videos and 88% of videos provided no transparency or contact information for further information or support. Privacy and accurate authorship disclosures were made in only 8% and 13% of the videos, respectively. Figure 3 shows these results in unsupervised hierarchical clustering, as well as the grouping by video category in Additional file 1: Fig. S1. Higher median HONcode scores were seen in videos produced by academic institutions or governmental organizations (median 3, range 2–6) and by independent medical professionals (median 4; range 2 to 6), compared to videos produced by news or media (median 2; range 0 to 2) and independent non-medical users (median 2; range 0 to 7) (p = 0.06). HONcode score was low for all videos provided by news/media (Table 3).

### LAM-related content score (LRCS)

**Fig. 4:**
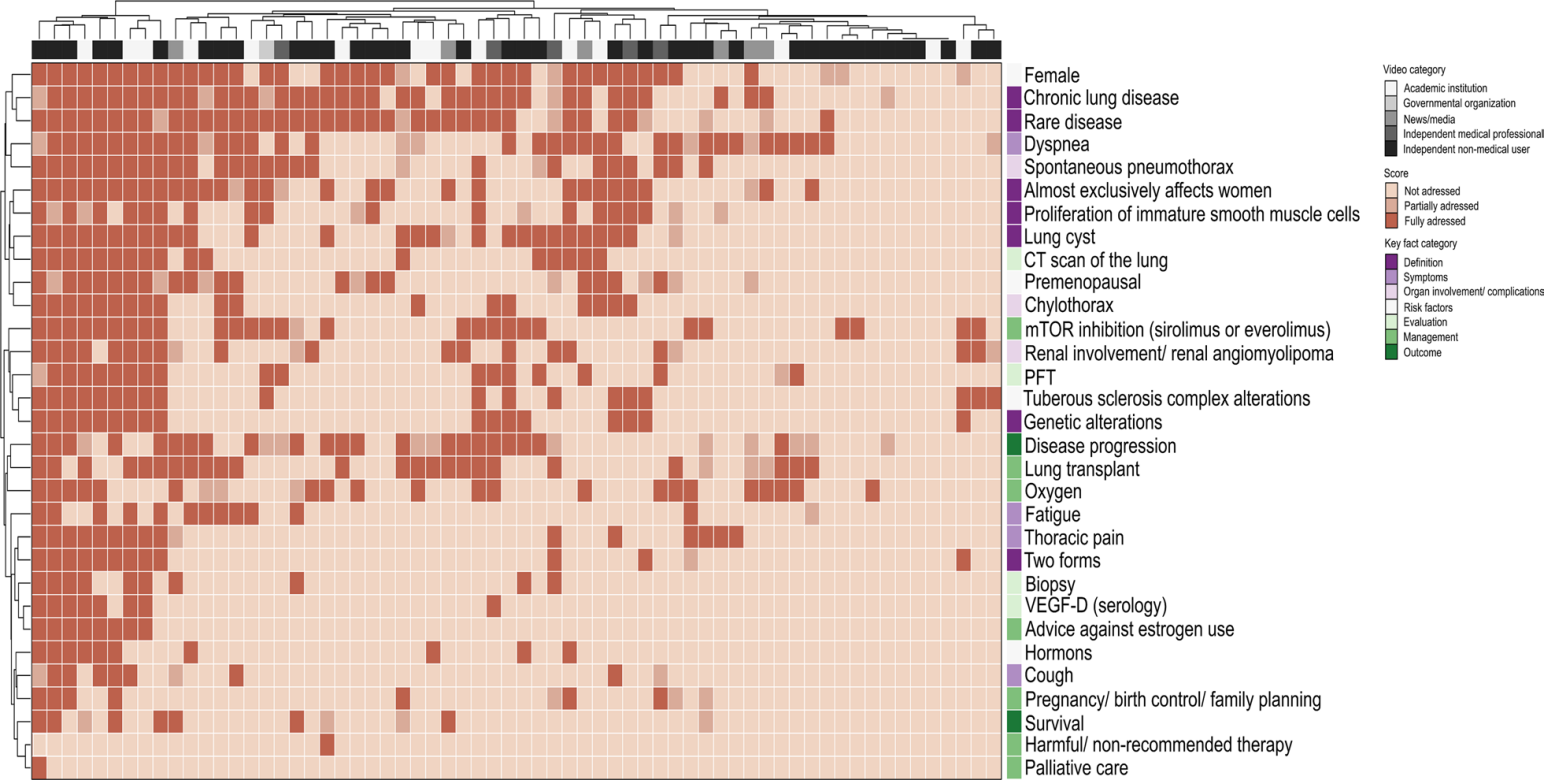
English videos content evaluation by LAM related content score. Unsupervised hierarchical clustering was performed by LAM related content score items (rows, n = 31) and single videos (columns, n = 64). In order to find potential clustering of video categories and key fact categories, the video category is shown in the top row (academic institution, governmental organization, news/media, independent medical professional, independent non-medical user) and the key fact item category (definition, symptoms, organ involvement/complications, risk factors, evaluation, management, outcome) corresponding to the single LRCS items in the last column of the heatmap. They are grouped into seven key fact categories, which are marked by different colours. The rating for each item (not addressed, partially addressed, fully addressed) is shown by intensity of brown colour (see score)

Videos mentioned a median of 8 (range 0 to 29) out of the 31 content items of the LRCS, corresponding to 26% of the essential information contained in the guidelines (Fig. 4). Most videos stated that LAM is a chronic lung disease (64%), is rare (64%), predominately affects women (64%), causes dyspnea (61%), and is progressive (52%) (Additional file 1: Table S3). Information on LAM pathophysiology, risk factors, and complications was often missing. Information related to diagnostic tests was provided in less than 30% of videos. mTor inhibitors were mentioned in 44% of videos, and lung transplantation in 42%. Recommendations related to pregnancy/family planning were included in 14% of videos. Two videos contained almost complete information and eight (24.8%) contained more than 22 key facts. Only 12% of videos were advising against estrogen use.

Comparison of videos across categories demonstrated a trend towards a higher median content score in videos provided by academic institutions or governmental organizations (median 9; range 0 to 25) and by independent medical professionals (median 10; range 8 to 13), compared to videos provided by news or media (median 7; range 4 to 15) or by independent non-medical users (median 6; range 0 to 29) (p = 0.19).

**Fig. 5:**
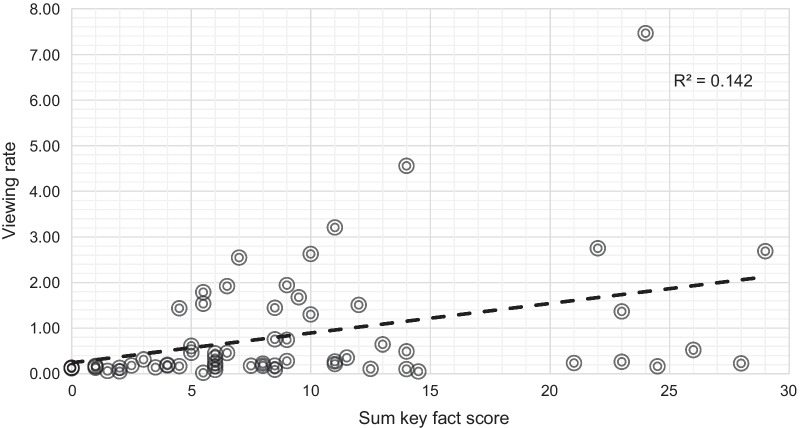
Unadjusted linear regression revealed a weak positive correlation between content score and viewing rate. One video with a viewing rate > 500 was excluded from the diagram to keep appropriate scaling

Unadjusted linear regression revealed a weak positive correlation between content score and viewing rate (R^2^ = 0.142, Fig. 5), but not initial search rank (R^2^ = 0.003, Additional file 1: Fig. S4), or engagement rate (R^2^ < 0.030), Additional file 1: Fig. S5a, b). Although videos with earlier search rank tended to have higher viewing rates, there was no association between search rank and viewing rate or engagement rate (R^2^ < 0.01, Additional file 1: Fig. S6a, b).

### Associations between general video characteristics and quality related scores

On multivariable linear regression, no explanation for the video ranking (model p value = 0.83) or viewing rate (model p value = 0.90) was found, when considering the HONcode score, sum DISCERN score, and content score as independent variables. There was a low (adjusted R^2^ = 0.0287) contribution of the content score, but not HONcode score or sum DISCERN score, to the engagement rate (model p value = 0.04). In models assigned constant values for the HONcode score and sum DISCERN score, an increase in the content score by one point was associated with an increase of the engagement rate of 0.006 (p = 0.01).

There was no specific information available on coronavirus infection for LAM patients (Covid-19) in any video 3 months after the first cases in Europe [21].

## Discussion

Our study is the first to investigate the content and quality of YouTube videos as a source of information for patients with LAM. Our data show that there is limited information available on YouTube for patients with LAM, with relevant differences in quality between the available videos that are unrelated to search rank or video source. Videos from independent medical professional tended to have a slightly higher HON foundation score, whereas News and media were consistently rated as “low”. Regarding the DISCERN score, highest rating was achieved for independent medical professional and lowest rating for news/media. Within the LRCS, videos from independent medical professional and academic/governmental institutions tended to perform slightly better compared to videos provided by news and media or by independent non-medical users.

We found considerable deficits in video quality scores by HONcode and DISCERN evaluation for all source types. The majority of videos had low to moderate adherence to HONcode principles, with only ten out of 64 videos (16%) having a high total HONcode score. This is similar to findings in a previous study of information on YouTube regarding IPF, another rare respiratory disease, where only 7% of videos reached a high score [14]. However, this contrasts with YouTube patient education videos regarding COPD, which demonstrated 69% of evaluate videos had high adherence to HONcode principles [22]. This could be due to the fact that there exists more information and relatively standardized action plans for common respiratory diseases like COPD [23] and asthma [24–26] than for rare lung diseases like LAM or IPF. Similarly, our evaluation of the DISCERN questionnaire, an instrument originally developed to evaluate the quality of written consumer health information on treatment choices [17], demonstrated that there are marked deficits in the quality of information presented on YouTube about LAM treatment. The majority of videos did not mention treatment options at all or lacked further information regarding patient support, sources of information, date of information, action plans, or risks of treatment, resulting in a poor average DISCERN that indicated poor overall quality (Table 3).

Video content was assessed using a newly created LAM-related content score (LRCS), which evaluated features of and therapies for LAM. On average, only 25% of all content items were mentioned in videos, and the majority of LAM-related content items were incompletely addressed. In a similar study evaluating patient education videos in IPF, only a median of 17% of content items were addressed [14], with non-recommended therapies being proposed as effective therapy in 17% of the videos. The slightly superior content of LAM-related videos compared to IPF-related videos may be related to the strong support network for patients with LAM over the past 25 years that has a very active patient advocacy group [27]. This may lead to better information dissemination regarding LAM compared to IPF, reflecting that IPF support groups are less well established and were founded later [28]. It is not possible, however, to measure the impact of the respective patient advocacy group, since the connection of a single video to the patient advocacy group cannot be clearly identified in most videos. It is also not possible to define the exact source of many YouTube videos, although we were able to document that 16 videos were posted by a patient advocacy group, while many others were from individual nonmedical users, who might still be members in a patient advocacy group.

The diagnosis of LAM can lead to considerable uncertainty and anxiety [29, 30]. The search for adequate, comprehensive information regarding LAM can be very time consuming. Most videos dealt with limited individual aspects of the disease, and we did not find any association between initial search rank and content score. The role of the YouTube algorithm in determining search rank is multifaceted. YouTube takes into account the search history, in addition the results for the same search queries may be different depending upon the user with non-transparent algorithms [31]. Depending on the personalized software with different operating systems (Windows, MacOS, IOS, Android, Linux, others) and hardware settings (PC tablet, smart phone, other devices) the user may get different results. It is not known if and how the search algorithm of YouTube is influenced by commercial interests e.g. of pharmaceutical industry to promote certain therapies for the disease. Search ranking as a complex process can be influenced by other content discovery and subscription features of the website [32]. The failure of this algorithm to preference quality videos has been identified as a problem, even despite high viewing rates quality videos can have a low search rank [32].

More suitable patient education videos may be found on the LAM-foundation homepage [33], but these videos will not be identified in a YouTube search as they are uploaded on Vimeo, an ad-free open video platform with a markedly smaller audience [34]. This highlights the need for comprehensive, accurate, and well-maintained resources for LAM-related patient information on YouTube and other social media platforms. *A comparison between YouTube and vimeo analysing the frequency of use and the content and quality of health information should be a topic for further research.*

Most evaluated videos were outdated, with only six uploaded in the year preceding analysis. The only available medical therapy for LAM, sirolimus, was approved by the U.S. Food and Drug Administration (FDA) in May 2015 and by the European Medicines Agency (EMA) in September 2018 [35, 36]. Twenty-four videos (38%) were uploaded before the FDA approval and 50 videos (78%) before the EMA approval [37], which may have contributed to the finding that treatment with mTor-inhibitors was not addressed in most videos.

Some limitations may apply to our study. Our data can only be applied to English speaking people and countries, where YouTube is available. YouTube’s search algorithm leads to user-dependent results in a dynamic system with inherent limitations such that each user may get different search results [31]. Since YouTube is not the sole video platform on the internet, inclusion of other platforms could have resulted in better scores; however, in this study we focused on the most frequently used video platform. Further studies should investigate the contribution of Vimeo, Facebook, Instagram, TikTok, and others social media platforms for a greater understanding of internet sources of patient information on LAM.

## Conclusion

The lack of knowledge about LAM as a rare disease is challenging for patients and physicians. LAM-related videos on YouTube frequently provide incomplete and low-quality information, especially regarding treatment options. Clinicians and patient advocacy organizations should collaborate to provide the best available sources of information for their patients with this rare disease.

## Supplementary Information

**Additional file 1.** Additional figures and Tables.

## Data Availability

All videos are in this study are freely available on www.youtube.com. The dataset of the individual videos with title, ranking and URL included can be found in Additional file 1. The datasets analysed during the current study are available from the corresponding author on reasonable request.

## Abbreviations

LAM: Lymphangioleiomyomatosis
HON: Health on Net
LRCS: LAM-related content score
EMA: European Medicines Agency
FDA: Food and Drug Administration
TSC-LAM: Tuberous sclerosis complex-associated LAM
IPF: Idiopathic pulmonary fibrosis
