# Supplementary material for: YouTube-videos for patient education in lymphangioleiomyomatosis?
Source: Respir Res. 2022 Apr 27;23:103. doi: 10.1186/s12931-022-02022-9 (PMC9043877; doi:10.1186/s12931-022-02022-9)
Supplement: Supplementary file 1 — Additional file 1. Additional figures and Tables. [file 12931_2022_2022_MOESM1_ESM.docx]

**Additional file 1**

**Table S1:** Overview of DISCERN instrument criteria for judging the quality of written consumer health information on treatment choices. For further details see reference (17).

**Section 1**

**IS THE PUBLICATION RELIABLE?**

1. Are the aims clear?

2. Does it achieve its aims?

3. Is it relevant?

5. Is it clear when the information used or reported in the publication was produced?

6. Is it balanced and unbiased?

7. Does it provide details of additional sources of support and information?

8. Does it refer to areas of uncertainty?

| No |  | Partially |  | Yes |
| --- | --- | --- | --- | --- |
| 1 | 2 | 3 | 4 | 5 |

**Section 2**

**HOW GOOD IS THE QUALITY OF INFORMATION ON TREATMENT CHOICES?**

9. Does it describe how each treatment works?

10. Does it describe the benefits of each treatment?

11. Does it describe the risks of each treatment?

12. Does it describe what would happen if no treatment

13. Does it describe how the treatment choices affect

14. Is it clear that there may be more than one possible treatment choice?

15. Does it provide support for shared decision-making?

| No |  | Partially |  | Yes |
| --- | --- | --- | --- | --- |
| 1 | 2 | 3 | 4 | 5 |

**Section 3**

**OVERALL RATING OF THE PUBLICATION**

16. Based on the answers to all of the above questions, rate the overall quality of the publication as a source of information about treatment choices:

| Low |  | moderate |  | high |
| --- | --- | --- | --- | --- |
| Serious or  extensive  shortcomings |  | Potentially important but  not serious shortcomings |  | Minimal  shortcomings |
| 1 | 2 | 3 | 4 | 5 |

**Table S2:** Brief details on the the eight HONcode criteria for medical and health websites. Further details accessed via <http://www.hon.ch/Conduct.html>.

| 1. **Authoritative** | Video provides a clear statement about qualifications of the author. |
| --- | --- |
| 1. **Complementarity** | Video intends to support, not replace, of the relationship between a patient and existing physician. |
| 1. **Privacy** | Video preserves confidentiality of data relating to individual patients. |
| 1. **Attribution** | Video provides clear references to source data and date of last modification. |
| 1. **Justifiability** | Video discusses the benefits or performance of a specific treatment, and provides appropriate, supporting evidence. |
| **6. Transparency** | Video provides contact information for viewers that seek further information or support. |
| **7. Financial disclosure** | Support for this Video is clearly identified. |
| **8. Advertising policy** | Any advertising is clearly identified or labelled. |

**Table S3:** Proportion of videos addressing each element of the LAM-related content score (LRCS).

| **Overall unique videos, n (%)** | **64 (100)** | | |
| --- | --- | --- | --- |
|  | **Fully addressed, n (%)** | **Partially addressed, n (%)** | **Not addressed, n (%)** |
| **Definition** |  |  |  |
| Two forms | 12 (19) | 1 (2) | 51 (80) |
| Chronic lung disease | 36 (56) | 5 (8) | 23 (36) |
| Proliferation of immature smooth muscle cells | 16 (25) | 6 (9) | 42 (66) |
| Lung cyst | 26 (41) | 2 (3) | 36 (56) |
| Rare disease | 35 (55) | 6 (9) | 23 (36) |
| Almost exclusively affects women | 28 (44) | 3 (5) | 33 (52) |
| Genetic alterations | 17 (27) | 0 (0) | 47 (73) |
| **Symptoms** |  |  |  |
| Fatigue | 12 (19) | 1 (2) | 51 (80) |
| Dyspnea | 32 (50) | 7 (11) | 25 (39) |
| Cough | 7 (11) | 3 (5) | 54 (84) |
| Thoracic pain | 15 (23) | 1 (2) | 48 (75) |
| **Organ involvements /complications** |  |  |  |
| Spontaneous pneumothorax | 26 (41) | 2 (3) | 36 (56) |
| Chylothorax | 18 (28) | 0 (0) | 46 (72) |
| Renal involvement/renal angiomyolipoma | 18 (28) | 4 (6) | 42 (66) |
| **Risk factors** |  |  |  |
| Female | 36 (56) | 5 (8) | 23 (36) |
| Premenopausal | 18 (28) | 8 (13) | 38 (59) |
| Tuberous sclerosis complex alterations | 19 (30) | 0 (0) | 45 (70) |
| Hormones | 10 (16) | 0 (0) | 54 (84) |
| **Evaluation** |  |  |  |
| CT scan of the lung | 17 (27) | 0 (0) | 47 (73) |
| VEGF-D (serology) | 8 (13) | 0 (0) | 56 (88) |
| PFT | 17 (27) | 2 (3) | 45 (70) |
| Biopsy | 10 (16) | 0 (0) | 54 (84) |
| **Management** |  |  |  |
| mTOR inhibition (sirolimus or everolimus) | 27 (42) | 1 (2) | 36 (56) |
| Lung transplant | 24 (38) | 3(5) | 37 (58) |
| Oxygen | 21 (33) | 3 (5) | 40 (63) |
| Palliative care | 1 (2) | 0 (0) | 63 (98) |
| Pregnancy/birth control/family planning | 7 (11) | 3 (5) | 54 (84) |
| Advise against estrogen use | 8 (13) | 0 (0) | 56 (88) |
| **Outcome** |  |  |  |
| Disease progression | 22 (34) | 11 (17) | 31 (48) |
| Survival | 7 (11) | 4 (6) | 53 (83) |

**Figure S1**: English videos content evaluation by HONcode score. HON foundation score items (columns) are presented for single videos (rows, n = 64). The HON principle criterium is either met or not met. The videos are grouped by video category. Rating according to HON foundation score: black = high, grey = medium, white = low

**Figure S2:**
DISCERN score items (columns) are shown for single videos (rows, n = 64). The categorial DISCERN item scoring ranges between 1 (not addressed) and 5 (fully addressed). The item 2. "aims achieved" was not assessable (NA) scores, when item 1. "explicit aims" was scored with 1, i.e., criterion not met. The videos are grouped by video category.

**Figure S3:** English videos content evaluation by LAM-related content score (LRCS). LRCS items (columns, n = 31) are shown for single videos (rows, n = 64). The content item scoring ranges between 0 (not addressed, white), 0.5 (partially addressed, grey) and 1 (fully addressed, black). The videos are grouped by video category.

**Figure S4:** Association between sum LAM-related content score and initial search rank.

Dashed line is the result of the linear regression model evaluating the association between search rank and LRCS


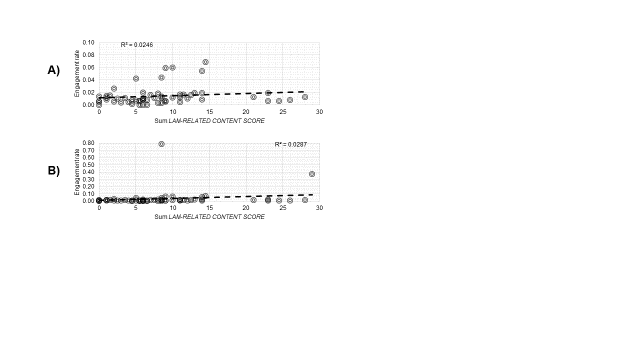


**Figure S5: A)** Association between sum LAM-related content score and engagement rate. Four videos were excluded from the diagram due to missing engagement rate data.

**B)** Association between sum LAM-related content score and engagement rate. Four videos were excluded from the diagram due to missing engagement rate data. Two additional videos with an engagement rate >0.1 were excluded from the diagram in order to keep appropriate scaling.


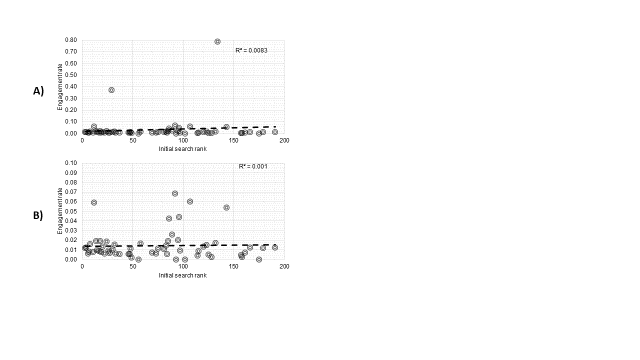


**Figure S6: A)** Association between initial search rank and engagement rate. Four videos were excluded from the diagram due to missing engagement rate data.

**B)** Association between initial search rank and engagement rate. Four videos were excluded from the diagram due to missing engagement rate data. Two additional videos with an engagement rate > were excluded from the diagram in order to keep appropriate scaling.

**Figure S7:** Association between initial search rank and viewing rate. One video with a viewing rate >500 was excluded from the diagram in order to keep appropriate scaling.
